# Conversational mHealth Platform Designed to Support Tuberculosis Treatment Adherence in Low-Income South African Patients: Pilot Cohort Study

**DOI:** 10.2196/85242

**Published:** 2026-07-08

**Authors:** Sean Richardson, Pamela Vorster, Ronelle Burger, Matthias Rieger

**Affiliations:** 1 Erasmus School of Health Policy & Management, Erasmus University Rotterdam, Burgemeester Oudlaan 50, Rotterdam, The Netherlands, 31 0629368273; 2 Department of Economics, Stellenbosch University, Stellenbosch, South Africa; 3 International Institute of Social Studies, Erasmus University Rotterdam, The Hague, The Netherlands

**Keywords:** tuberculosis, treatment adherence, mHealth, WhatsApp intervention, chatbot, conversational platform, South Africa, pilot study, low- and middle-income countries, behavioral economics, mobile phone

## Abstract

**Background:**

Tuberculosis is a leading cause of death in South Africa, with poor adherence undermining treatment success. Findings from recent research on the impact of mHealth (mobile health) interventions on tuberculosis treatment outcomes show promise, yet many interventions remain untested in African contexts. Rising smartphone ownership in South Africa enables more complex mHealth interventions, offering an opportunity to deploy behavioral tools within high-burden, resource-constrained settings.

**Objective:**

This pilot study evaluates the feasibility and effectiveness, among low-income patients at a South African clinic, of a WhatsApp (Meta)-based conversational mHealth platform designed to tackle specific behavioral barriers to adherence. Aims include the following: (1) evaluating coverage by studying the proportion of patients within the target group who own smartphones, (2) describing patterns of engagement with the platform and the role of mobile data scarcity as a barrier to use, and (3) producing evidence on the impact that a behavioral mHealth intervention can have on tuberculosis treatment success.

**Methods:**

Patients newly diagnosed with drug-susceptible pulmonary tuberculosis between August 2022 and October 2023 completed a screening survey. Those owning compatible mobile phones were invited to enroll. The platform provided reminders alongside behavioral support features. Coverage was studied by estimating smartphone ownership among screened patients and comparing characteristics between enrolled patients (n=42) and those receiving standard care (n=102) using standardized differences. Engagement was analyzed using local polynomial regressions for usage trends and logistic regressions to estimate the impact of mobile data top-ups. The marginal effect of enrollment on the probability of successfully completing tuberculosis treatment was studied using a *t* test and logistic regressions with and without covariates.

**Results:**

A total of 34% (49/146) of screened participants owned a phone that could use WhatsApp. There were differences in characteristics by enrollment status. Further, 50% of patients engaged with the platform each day until the end of treatment. Overcoming an initial inability to send unprompted messages to inactive patients was associated with an immediate 13-percentage-point increase in aggregate engagement the following month. Mobile data scarcity hindered use—receiving mobile data top-ups within the previous week was associated with a 3.37-percentage-point increase (95% CI 0.0007 to 0.0666) in platform engagement. The estimated marginal effect of enrollment was a 17.6-percentage-point (95% CI 0.003 to 0.348) increase in treatment completion, becoming attenuated after adjusting for patient characteristics (12.8 percentage points, 95% CI −0.048 to 0.304).

**Conclusions:**

While phone ownership and mobile data constraints represent barriers to feasibility, findings suggest that smartphone-based mHealth interventions may aid successful treatment completion—alleviating health system burdens by automating care for less vulnerable patients. Engagement with the platform throughout tuberculosis treatment was high and stable, and enrolled users experienced a higher success rate. A randomized controlled trial is required for impact evaluation.

## Introduction

### Background

Inadequate medication or treatment adherence remains one of the costliest inefficiencies in health care systems worldwide [1]. As smartphone ownership rates are rising in low and middle-income countries (LMICs), mHealth (mobile health) interventions relying on platforms such as WhatsApp (Meta) coupled with behavioral insights could offer new opportunities to tackle persistent problems with medication adherence. Evidence on the impact of treatment adherence mHealth interventions in LMICs remains mixed [2,3]. There have been calls for more research on this topic, and in particular, research that can enhance the understanding of implementation problems and how to ensure that platforms are patient-responsive [4,5].

### Tuberculosis in South Africa

Despite the availability of effective diagnosis and cure, tuberculosis is the leading cause of death due to infectious disease in the world [6]. In South Africa, the estimated tuberculosis incidence was 249,000 in 2024, with a 23% fatality rate, surpassing the global fatality rate of 12% [6].

Treatment of drug-susceptible tuberculosis is highly effective but requires 6 months of daily oral medication. Treatment challenges in South Africa are compounded by a dual burden of tuberculosis and HIV. Around 1 in 5 adults in South Africa lives with HIV. Among these, about 20% are not on antiretroviral therapy, and another 20% are not virally suppressed [7]. Tuberculosis progresses faster and is more fatal among people with uncontrolled HIV [8,9], which significantly heightens the risks associated with late tuberculosis diagnosis and poor treatment adherence.

South Africa’s treatment success rate averaged 71% in 2023, well below the global average of 88% and the World Health Organization target of 90% [6,10]. Patients with tuberculosis are a vulnerable subpopulation and disproportionately drawn from the bottom of the income distribution. Nonadherence to tuberculosis treatment remains a major barrier to disease control, driving higher tuberculosis incidence, the spread of drug-resistant strains, and poor treatment outcomes [11].

### The Role of Digital Health Interventions

There is a growing interest in the use of mHealth technology to improve the outcomes of patients undergoing treatment for tuberculosis. Lee et al [12] systematically evaluated the methodologies of 27 digital interventions to boost tuberculosis treatment adherence between 2012 and 2022, of which 70% (19/27) were published in 2019 or later. They note that this uptick in academic interest coincides with a move away from simple SMS reminders in favor of electronic, directly observed therapy, smart pillboxes with built-in adherence tracking and reminders, and broader mHealth interventions. Importantly, this review notes that the efficacy of two-way platforms allowing patients to interact with a health care provider or representative agent had been underexplored by that point.

In terms of the impact of digital adherence technologies on tuberculosis treatment outcomes, Mohamed et al [13] conducted a comprehensive systematic review and meta-analysis of 76 studies published between 2002 and 2024. Despite moderate success overall (odds ratio=1.14, 95% CI 0.99 to 1.30), the outcomes of these interventions are inconsistent and highly heterogeneous. This study also highlights a lack of evidence regarding two-way digital health platforms. Concerning specific interventions, smartphone-based and video-based directly observed therapy interventions show the most consistent promise, although the settings of these studies impact generalizability, with none of the reviewed video-based directly observed therapy interventions taking place in LMICs and none of the reviewed smartphone-based interventions taking place in Africa. The interventions studied in LMICs have relied on SMS reminders, medication sleeves, or smart pillboxes, all of which have shown mixed results. Adding to this discussion, a major recent study consisting of four cluster-randomized trials in the Philippines, South Africa, Tanzania, and Ukraine showed limited evidence regarding the potential for smart pillboxes to improve treatment outcomes [2].

The range of possible digital health interventions in high-burden countries has expanded massively in recent years. In 2023, an overwhelming majority of South Africans used the internet (78%), owned a smartphone (71%), and used social media (71%) [14]. Promising interventions that were considered only feasible in higher-income societies with comparatively low tuberculosis burdens can now be tested in settings where they stand to generate the greatest societal benefit.

### Behavioral Foundations of Nonadherence

Medication nonadherence is—broadly speaking—either rational or behavioral in nature [15]: Rational patients evaluate costs and benefits of medication adherence using all information available to them. Costs are typically immediate (eg, side effects, hassle, and travel), while benefits (eg, cure and productivity gains) occur in the future and are exponentially discounted. In the case of tuberculosis, the eventual benefits of adhering to treatment are very large relative to costs. Nonadherence can be fatal. While the medication does have immediate side-effects, chances of cure are sizeable and, in South Africa, it can be collected for no direct cost at public clinics. Behavioral risk factors influence this rational cost-benefit calculation in systematic and predictable ways [16]. Patients could be present-biased, putting a very large weight on the present and immediate gratification. For example, patients may heavily overweigh the side effects at the time of taking medication. They may also misperceive the costs and benefits of treatment. For example, they might misjudge the effectiveness of the medication. Both time inconsistency and misperceptions, in turn, lower adherence. Medication adherence also requires a daily discipline and routine; forgetfulness can lead to unintentional interruptions of the treatment regimen. Behavioral dimensions relating to stigma and the lack of psycho-social support during a long and taxing treatment journey can simultaneously have a direct impact on patient behavior and compound the impact of other behavioral factors.

### Study Objectives

We respond to South Africa’s tuberculosis treatment adherence problem by designing a novel WhatsApp-based support platform targeted at drug-susceptible tuberculosis patients from low-income communities. We used basic behavioral economic insights to design our adherence platform, aiming to motivate, support, inform, and remind patients over the treatment journey [17] with design choices that were expressly mindful of scalability and potential for broader implementation. We combined components of text-based reminders and broader mHealth interventions, delivering these interventions through a conversational platform that also lays the groundwork for research into two-way functionality as digital tools continue to evolve.

This paper reports practical insights from a year-long pilot of this mHealth intervention in one clinic in the Western Cape. This pilot study has several main aims. First, we aim to evaluate coverage by understanding the proportion of patients within the target group who own smartphones and can thus be targeted by such an intervention. Second, we seek to understand usage of the chatbot by describing patterns of engagement, including an analysis of mobile data shortages as a barrier to usage. Third, and arguably most important, we aim to produce suggestive evidence regarding the impact that a behavioral mHealth intervention can have on treatment success among patients with tuberculosis in a low-income setting by comparing outcomes among those enrolled on the platform to those experiencing standard of care. With a rapidly evolving field, offering evidence as to the merit of these interventions is important in ensuring that this work receives the attention that it deserves. Additionally, by providing a thorough overview of operational challenges experienced, we aim to help future studies avoid pitfalls and maximize the societal benefit of their work.

This pilot is also intended as a proof-of-concept, with findings used in the design of a larger individual-level randomized controlled trial (Pan African Clinical Trial Registry, PACTR202501746484460) studying the impact of this intervention in 10 clinics across the Western Cape. Data collection for this study is expected to be completed in 2027.

## Methods

### Design of a Chatbot Platform to Support Tuberculosis Treatment Journeys Among Low-Income Patients

**Figure 1.**
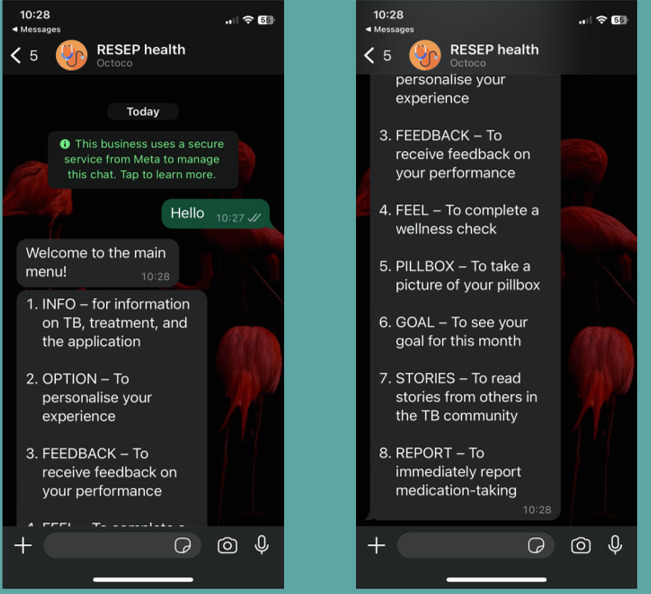
Example screenshots from conversational mHealth platform. RESEP: Research on Socioeconomic Policy; TB: tuberculosis.

Our WhatsApp-based conversational platform was developed using insights from psychology and behavioral economics, incorporating gamification elements to foster medication adherence and support sustained behavior change. Participants received daily medication reminders (sent at a time of their choosing), which prompted them to report whether they had taken their medication, leveraging salience to reduce forgetfulness. Additionally, patients were prompted to respond to health-related check-ins and periodically complete quizzes to reinforce knowledge about tuberculosis transmission and treatment. Patients received congratulatory messages and within-platform “points” for engagement, providing frequent intangible rewards for continued interaction with the intervention. The platform enabled limited two-way interaction: patients could respond using a structured menu, and the system provided encouraging feedback and recognition when prompts were answered or milestones achieved. Patients could also submit photos of their pill boxes to monitor medication collection. The screenshot shown in Figure 1 presents the main menu through which patients could access the features of the platform. To further encourage adherence and offset immediate, perceived costs of adherence, participants received microrewards in the form of WhatsApp data for meeting predefined targets for reporting pill-taking.

Given that this study targeted a vulnerable subpopulation of poor South Africans living with tuberculosis, the design of the conversational platform was deliberately iterative to better respond to patients’ needs and barriers to engagement. The fieldworker actively sought feedback from platform users during their clinic visits and supplemented this with follow-up phone calls to understand their experiences and challenges. Insights from this feedback, together with our analysis of patterns of patient engagement on the platform, informed successive rounds of improvements. Table 1 summarizes these changes, which included translating the platform into Afrikaans and isiXhosa (the platform was initially designed in English, which, while widely understood and spoken in the setting, is generally spoken as a second or third language), introducing quizzes to make the content more engaging, developing an SMS-based version for patients without internet-enabled phones, and implementing push notifications to re-engage patients after periods of inactivity.

Push notifications were introduced shortly after WhatsApp updated its customer interaction policies to permit this functionality. Initially, due to WhatsApp’s antispam measures, we were only able to send messages to patients who had interacted with the conversational platform within the previous 24 hours. As a result, patients who stopped reporting medication adherence ceased to receive messages until they proactively reinitiated contact. From February 2023 onward, however, the platform began sending preapproved push notifications to inactive patients, enabling continued outreach beyond the 24-hour window.

Participants received monthly WhatsApp data bundles to ensure that they had data to engage with the platform. They received approximately one gigabyte per month, with some variation across networks. The monthly WhatsApp data bundles were sent directly to the phones of participants.

**Table 1.** Date of intervention and changes made to the conversational mHealth platform.

Date	Change to the conversational platform
August 2022	Start screening survey (English, Afrikaans, and isiXhosa)
September 2022	First enrollment on conversational platform (English only)
February 2023	Rollout of conversational platform in Afrikaans and isiXhosa
February 2023	Gained ability to send prompts to participants to re-engage after a period of nonengagement
March 2023	Introduce quizzes and social nudges
March 2023	Rollout of SMS version
August 2023	Introduce personal goal setting

### Participant Recruitment, Data Collection, and Study Design

This pilot study took place at a small public clinic in Groendal, just outside the town of Franschhoek in the Western Cape province. Fieldwork commenced in August 2022 with a short pilot of the patient flow and screening processes taking place before the enrollment window opened. The first patient was enrolled on the platform on September 2, 2022. Patient enrollment and primary data collection concluded on October 25, 2023, with the exception of treatment outcomes, which were captured using secondary data from the clinic register and nurse logs. This time window was not predetermined and primarily closed due to resource requirements for the setup of the full-scale experiment. Although data collection closed at this time, the platform remained functional for several more months. The last treatment outcome recorded occurred in April 2024.

We recruited and trained a fieldworker to screen and enroll newly diagnosed patients with tuberculosis at the clinic. Note that the fieldworker is from the local community, was recommended by clinic staff, and was recruited, paid, and placed at the clinic by RB (coprincipal investigator). Patients were directly referred to the fieldworker by the tuberculosis nurse on duty, after which they were screened for eligibility. Individuals were eligible to participate in the data collection if they were aged 18 years or older, were literate (able to read and respond to messages), and had received a new diagnosis of drug-susceptible pulmonary tuberculosis within the last two months. Screening was not possible for patients not referred by the nurse (including cases in which the fieldworker was unavailable) or patients who declined to meet with the fieldworker. Patients are included in the analysis if they were properly screened, provided informed consent, and their medical eligibility could be confirmed using the clinic register. Screening, elicitation of consent, and the baseline survey were conducted on a tablet connected to the internet, which was kept in the fieldworker’s office.

The baseline survey included questions on socioeconomic status, demographics, mobile phone usage, access to health care facilities, and tuberculosis knowledge. All nontreatment patient characteristics used in this study were obtained from this survey. This survey was conducted using SurveyCTO on the aforementioned secure tablet.

Eligible participants who indicated possession of a phone with WhatsApp installed were invited to sign up for the conversational platform. All patients not onboarded onto the intervention, including patients screened in the month between the commencement of data collection and the rollout of the platform, received the standard of care. The eligibility criteria allowing onboarding within the first two months of treatment mean that patients screened from the month before the rollout are a comparable cohort. Modified eligibility criteria for an SMS-based version that did not require smartphone ownership were also piloted.

Performance of the platform, including message delivery and reported pill-taking (defined below), was evaluated using high-frequency data from the chatbot servers during the primary collection period, which was provided to the authors by the development team. Receiving mobile data bundles was incorporated into engagement analysis using information accessed through SIMcontrol, which was used to send these bundles.

A supplementary round of data collection was conducted from August to November 2023, consisting of 15 in-depth qualitative interviews conducted in a private venue in a local community center in Groendal. This process was not led by the authors of this paper, and the outcome of this process is the focus of an additional manuscript awaiting publication.

### Ethical Considerations

This study was approved by the Health Research Committee of Stellenbosch University (reference M21/08/016). Only patients over the age of 18 years were eligible to join this study. All participants could therefore sign the consent on their own. In addition to the consent form being made available in English, Afrikaans, and isiXhosa on the tablet, patients received a hard copy. The field worker explained the consent form and this study’s risks to participants and answered any questions they may have had. The English version of the consent form is included as Multimedia Appendix 1.

Information was only shared with authorized study staff. Individual identifiers were required for matching participants across multiple datasets, including the screening surveys, the platform engagement data, and routine data from the clinics. After merging data sources, the data were deidentified for the analysis.

All participants received a small gift to thank them for completing the short screening survey. Participants who were eligible for this study received a monthly data allowance to cover the data costs from participating in this study.

### Analysis

#### Sample Composition and Smartphone Ownership

We report a breakdown of the sample, starting with the total number of patients considered medically eligible to participate in this study as per the clinic register. We present how the screening and onboarding processes generated the sample sizes used in the analysis, with a specific focus on the role of smartphone ownership in eligibility.

#### Patient Characteristics

We present and compare socioeconomic and demographic characteristics of the enrolled and unenrolled samples to gain insight into factors that may confound the observed association between platform enrollment and treatment success. The variables presented reflect participants’ sex, age, education, and employment status. Table 2 provides comprehensive information on these characteristics, along with all other variables examined in this study. Standardized differences in characteristics between groups are reported as Cohen *d* values for comparability and ease of interpretation.

**Table 2.** Summary of outcomes, treatment conditions, and covariates used in quantitative analysis.

Variable	Description
Outcomes	
Treatment success	Binary variable. Defined as either bacteriological evidence of cure (negative sputum test at endline) or programmatic success as per clinic records (not missing more than two consecutive monthly pill collections). Missing if medically ineligible or transferred out.
Engagement with platform	Binary variable. Any reported pill-taking behavior (positive or negative) on a day. Missing for patients not enrolled. Patients with no documented engagement (n=3) omitted from analysis.
Delivery of messages	Binary variable. Any message from the chatbot successfully delivered to the user on a given day. Missing for patients not enrolled. Patients with no documented engagement (n=3) omitted from analysis.
Treatment conditions	
Enrollment on chatbot platform	Binary variable. Equals one if a patient was enrolled on chatbot, zero otherwise. Missing values for all unscreened or medically ineligible patients.
Receiving mobile data in last week	Binary variable. Equals one if a patient was sent mobile data as part of involvement in study during the week before the observed date, and zero otherwise. Missing for patients not on the platform.
Covariates	
Sex	Binary variable reflecting patient sex as per the clinic register. Used in treatment outcome analysis.
Age	Binary variables reflecting age brackets. Used in treatment outcome analysis.
Education	Binary variables reflecting the highest level of educational attainment. Used in treatment outcome analysis.
Employment	Binary variables reflecting current employment status. Used in treatment outcome analysis.
Individual fixed effect	Categorical variable assuming a different outcome for each user, used to control for between-user variation. Used in analysis of impact of mobile data top-up on engagement.
Calendar month	Categorical variable reflecting which month of this study the observation occurred in. Used in analysis of impact of mobile data top-up on engagement.
Month of journey	Categorical variable reflecting which month of the journey (with users starting at month 0 and offboarding in month 6) the user falls in on the specific date. Used in analysis of impact of mobile data top-up on engagement.
Day of week	Categorical variable reflecting the day of the week. Used in analysis of impact of mobile data top-up on engagement.

#### Platform Engagement and Data Access

We gauge user engagement for all patients successfully enrolled on the platform via self-reported medication-taking (any reporting, either positive or negative) each day during their treatment. Negative reports are included as they still reflect user engagement with the platform’s most salient feature, and to prevent engagement from directly reflecting treatment adherence rather than engagement. We also present the share of patients successfully receiving messages each day over time as context for the impact of technical challenges faced by the platform on engagement. First, we present the values for engagement and delivery averaged over each calendar date. Second, we present average daily engagement figures over the duration of the individual treatment journey, differentiated by whether the individual was onboarded before or after January 1, 2023. This date has been chosen because (1) it produces approximately equal subgroups and (2) a gap in enrollment between November 1, 2022, and January 9, 2023, makes these cohorts distinct. Both sets of results are presented as the output of local polynomial regressions with a span parameter of 0.2, meaning that the predicted value is estimated using a bandwidth that contains the nearest 20% of the sample.

We assessed the importance of data access by comparing platform engagement between patients who had received their monthly mobile data top-ups within the last week and patients who had not. We estimate this using a simple logistic regression model without time and individual fixed effects (model 1), a panel logistic regression including individual and time fixed effects (model 2), and a model with the same fixed effects estimated only for the cohort enrolled after January 1, 2023 (model 3). For each model, we present the estimated average marginal effects of recently receiving data. The model with fixed effects aims to isolate the impact of the top-up by controlling for patient- and time-based variation in engagement with the chatbot. The time fixed effects used in the conditional models include calendar month, month of individual user journey, and day of week of the observation. The cohort-specific model is used to test validity by estimating the impact of receiving data during the period in which engagement is less affected by technical issues.

For both analyses in this subsection, patients who never sent a single message on the platform (n=3) are excluded. Due to the use of individual-level fixed effects, the conditional models exclude three users who had 100% engagement with the platform during the observed time window.

#### Successful Tuberculosis Treatment

We examine the impact of the pilot on clinical outcomes using routine data on successful treatment outcomes, compared between patients enrolled on the platform and those receiving standard care. A patient’s treatment is categorized as a success in the clinic register if there is evidence of either cure (negative sputum results at 5 months) or successful completion of treatment (not missing more than 2 consecutive monthly pill collections). We compare means with a 2-tailed *t* test with robust SEs. We estimate the association between enrollment in the conversational platform and outcomes using two logistic regression specifications: an unadjusted model without covariates (model 4) and a model adjusting for all baseline participant characteristics presented in the comparison between treatment arms (model 5). The adjusted model aims to remedy potential nonrandom selection into treatment by controlling for the impact that observed characteristics may have on treatment outcomes. The results are reported as average marginal effects.

#### Software Used

Statistical analyses were conducted using R (version 4.4.0; R Foundation) and Stata (version 19.5; StataCorp LLC). Data cleaning, along with the estimation and visualization of local polynomial regressions, was performed in R. All effect estimates were produced in Stata. All software was run on the Windows 11 (Microsoft Corp) operating system.

## Results

### Sample Composition and Smartphone Ownership

**Figure 2.**
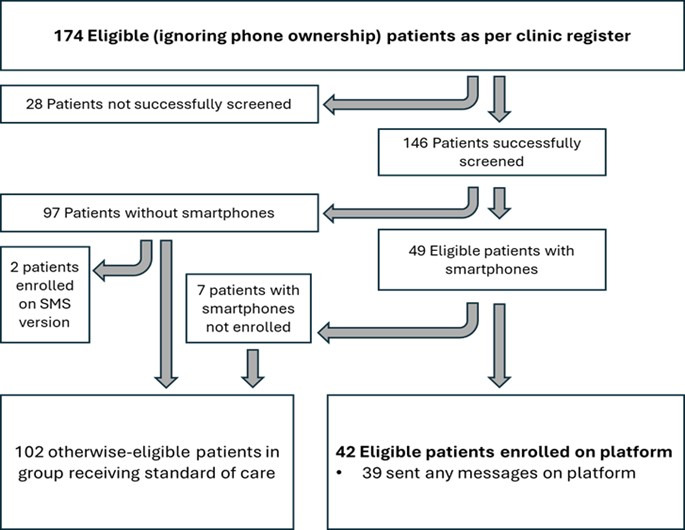
Sample composition–patients successfully screened, patients with smartphones, and patients enrolled on conversational platform, as compared to patients with tuberculosis considered eligible as per clinic register.

Figure 2 provides an overview of the data flow from eligible patients to the subsample registered on the WhatsApp platform. From the clinic register, 174 patients were identified who met the eligibility criteria of being aged 18 years or older and having new diagnoses of pulmonary tuberculosis. Of these patients, 146 patients were successfully screened by the fieldworker, of whom 49 patients were eligible for enrollment on the treatment support platform due to smartphone ownership. Based on this, the estimated rate of ownership of a phone with WhatsApp capabilities of the 146 screened participants was 34%. A total of 7 patients with smartphones were screened but not enrolled; 4 patients were screened before onboarding was fully functional, but during the inclusion period, and 3 patients were screened once onboarding was functional but were not successfully enrolled. Only 2 patients without smartphones signed up for the SMS-based version following its introduction—these patients are excluded from the sample for the remainder of the analysis.

The resulting analysis sample consists of two groups. First, 42 eligible patients enrolled on the WhatsApp platform. Second, the 102 eligible patients receiving standard care comprised of the 95 patients without smartphones who were not enrolled on the SMS platform and the 7 unenrolled patients with smartphones.

### Patient Characteristics

Table 3 compares the baseline demographic and socioeconomic characteristics of participants enrolled on the platform with those receiving standard care. Female participants were overrepresented in the enrolled sample by 7 percentage points. Patients with smartphones tend to be younger, on average. Additionally, enrolled patients are substantially (41 percentage points) more likely to have completed secondary schooling, and moderately (15 percentage points) more likely to be employed. The 7 smartphone-owning patients not enrolled on the platform (Multimedia Appendix 2) more closely resemble those receiving standard care than enrolled patients, particularly in terms of educational attainment and employment, suggesting that their exclusion is unlikely to systematically alter the characteristics of the standard of care group.

**Table 3.** Descriptive statistics of patients with tuberculosis group enrolled on conversational platform compared to those receiving standard care.

Variable and category	Enrolled on platform, n (%)	Standard of care, n (%)	Standardized mean difference
Sex			
Male	22 (52.38)	61 (59.80)	−0.15
Female	20 (47.62)	41 (40.20)	0.15
Age (years)			
18‐29	11 (26.2)	22 (21.57)	0.11
30‐39	15 (35.71)	31 (30.39)	0.11
40‐49	12 (28.57)	20 (19.61)	0.22
50‐59	4 (9.52)	21 (20.59)	−0.29
60+	0 (0.00)	8 (7.84)	−0.35
Education (highest level attained)			
Primary or less	4 (9.52)	17 (16.67)	−0.20
Incomplete secondary	12 (28.57)	64 (62.75)	−0.72
Completed secondary	24 (57.14)	19 (18.63)	0.91
Any tertiary (complete/incomplete)	2 (4.76)	2 (1.96)	0.17
Employment			
Employed	27 (64.29)	50 (49.02)	0.31
Unemployed, not looking for work	2 (4.76)	14 (13.73)	−0.29
Unemployed, looking for work	13 (30.95)	38 (37.25)	−0.13

### Platform Engagement and Data Access

**Figure 3.**
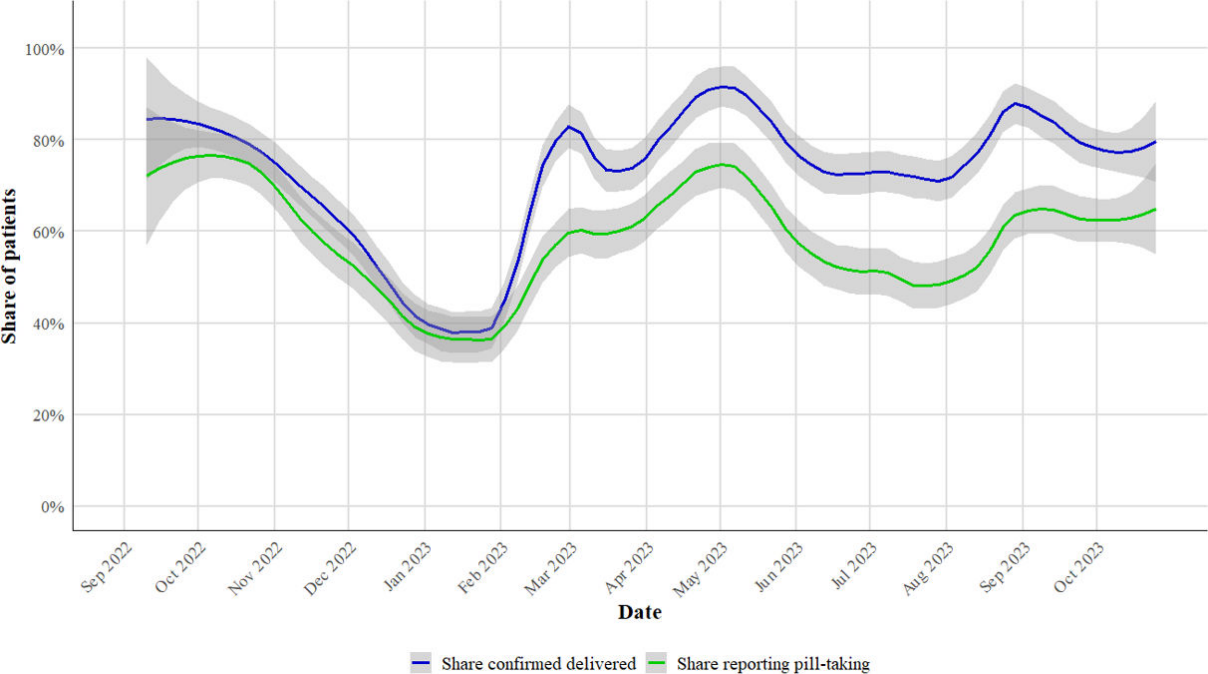
Daily share of patients receiving messages (blue) and reporting pill-taking behavior (green) over this study’s period. Trends modeled using local polynomial regression (span=0.2). Shaded areas represent 95% CIs.

Figure 3 compares the share of participants who received messages (blue) and those who reported their medication taking (both as adherent and nonadherent) over time (green) across this study’s period. The blue line is below one, indicating that not all messages were delivered. We observe a decline over time in both delivery and engagement, followed by a steep increase in February; average daily engagement fell to 37.2% in January, with a rise to 50.3% the following month. The figure illustrates that message delivery and engagement track each other closely throughout.

**Figure 4.**
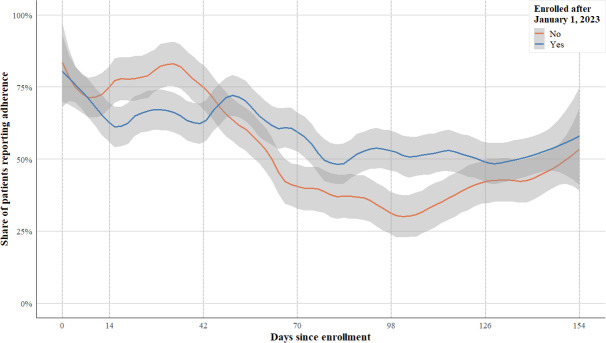
Aggregated daily engagement with the conversational platform over the tuberculosis treatment journey. Trends modeled using local polynomial regression (span=0.2). Shaded areas represent 95% CIs. Comparison shown between patients enrolled before (orange) and after (blue) January 1, 2023.

Figure 4 illustrates the patients reporting medication-taking from the start of enrollment throughout the approximately five and a half months of treatment, differentiated by start date. The pronounced lines at days 14, 42, 70, 98, 126, and 154 indicate the dates when medication is scheduled for collection, assuming a patient enrolls at diagnosis. In both groups, we observe that average engagement starts at approximately 80% and slowly declines over the treatment journey. The cohort that enrolled after January 1, 2023, experienced a more gradual decline in engagement, with long-term engagement stabilizing at a higher level than in the earlier cohort. We note that a significant drop-off among patients in this later cohort occurs before day 70 (third pill collection), particularly within the first month and a half of enrollment on the platform. After this point, engagement remains steady, with approximately 50% of users reporting medication-taking each day.

Among the patients on the SMS-based version of the platform, who are not included in this analysis, 1 patient had a 93% daily engagement rate aggregated over the entire journey, while the other had a 7% engagement rate.

Table 4 shows that receiving a data top-up in the previous week was significantly associated with a higher likelihood of engagement (model 1, average marginal effect=0.153, 95% CI 0.125 to 0.180, *P*<.001). This association remains after controlling for time and individual fixed effects (model 2, average marginal effect=0.0337, 95% CI 0.0007 to 0.0666, *P*=.045), although the point estimate is attenuated. The association is stronger among patients enrolled after January 2023 (model 3, average marginal effect=0.105, 95% CI 0.041 to 0.168, *P*=.001), who were less constrained by message sending limitations.

**Table 4.** Marginal effects of receiving a mobile data top-up in the previous week on the probability of daily engagement with the conversational platform during tuberculosis treatment estimates from logistic regressions.

	Model 1: daily engagement—without time or individual fixed effects	Model 2: daily engagement—with time and individual fixed effects	Model 3: daily engagement—fixed effects, post-January 2023 enrollment
Marginal effect of mobile data top-up in the last week on the probability of engagement on a given day (95% CI)	0.153 (0.125 to 0.180)	0.0337 (0.0007 to 0.0666)	0.105 (0.0417 to 0.168)
Time fixed effects: calendar month, month of journey, day of week	No	Yes	Yes
Individual fixed effects	No	Yes	Yes
N	4401	4356	2881

### Treatment Success

Comparing treatment outcomes between groups, we observe a significantly (*P*=.02) higher treatment success rate among those on the mHealth platform (90.48%) compared to those in standard of care (75.49%). Table 5 presents the marginal effects of the intervention, with the corresponding CIs, estimated using an unconditional logistic regression (model 4) and a logistic regression including the full list of covariates reported in Table 3 (model 5). The estimated average marginal effect of enrollment is 18 percentage points (95% CI 0.003 to 0.348, *P*=.046) in the unconditional model. The inclusion of the covariates in model 5 slightly reduces the magnitude of the estimated effect and results in the estimate no longer being significant at a 5% level.

**Table 5.** Marginal effects of enrollment on the chatbot platform on the probability of patients with tuberculosis successfully completing treatment—estimates from logistic regressions.

	Model 4: treatment success—no adjustment for participant characteristics at baseline	Model 5: treatment success—adjustment for participant characteristics at baseline
Marginal effect of enrollment on treatment success rate (95% CI)	0.176 (0.003 to 0.348)	0.128 (−0.048 to 0.304)
Covariates (all variables in Table 3)	No	Yes
N	144	144

## Discussion

### Principal Findings

This study had several aims. First, it sought to evaluate coverage by understanding the proportion of medically eligible patients that can be targeted by such an intervention. Only 34% (49/146) of screened individuals reported that they owned a mobile phone that was able to access WhatsApp, pointing to this intervention only being able to target a portion of patients with tuberculosis in this setting. The second aim of this study was to describe patterns of engagement, with a particular focus on the role of mobile data shortages as a barrier to use. We found evidence that most patients kept engaging with the platform throughout the duration of the treatment journey; daily engagement rates, measured by the rate of response to pill-taking prompts, started at approximately 80% at treatment start and stabilized at approximately 50% after several months among patients enrolled after January 1, 2023. Lastly, evidence points to mobile data being an important barrier to usage. Users who received a top-up in the previous week were found to be 3.37 percentage points more likely to engage with the platform, with this estimated effect rising to 10.5 percentage points by restricting the sample to the later cohort. Third, a key objective of this study was to provide evidence on whether enrollment on the chatbot platform was associated with better treatment outcomes, measured by binary treatment success (either bacteriological or programmatic). The estimated average marginal effect of enrollment was 18 percentage points, representing a large increase in the probability of completing treatment relative to other mHealth studies. Controlling for patient characteristics resulted in the estimated effect being somewhat attenuated and losing significance.

We posit that the lower-than-anticipated smartphone ownership is due to a combination of factors. These factors include the rural location of the clinic [18], tuberculosis primarily affecting poorer individuals [19] who are less likely to own smartphones [20], and the restrictive nature of conditioning eligibility on individual phone ownership rather than access to a shared phone [21]. Our initial assumption was that lower smartphone ownership was driven by patients predominantly using phones that could not access WhatsApp. We were only able to enroll 2 patients into the SMS version; however, suggesting that this was not a primary barrier. Our initial expectations regarding smartphone ownership were driven by conversations with individuals largely working in urban or periurban settings and the results of several studies indicating high rates of smartphone ownership and technological literacy among patients with tuberculosis, such as one study reporting 90% phone access among clinic patients in the urban setting of Matlosana/Klerksdorp [22]. On the other hand, the rate of phone ownership found in this study is similar to that found in a comparable study in Khayelitsha in the Western Cape [23]. For future studies, researchers may choose to overcome this by specifically targeting clinics in more urban settings. Another option is to expand eligibility to individuals with access to shared phones, although this raises privacy concerns if an individual does not wish for information relating to their tuberculosis status to spread.

We interpret the initial drop-off at the start of the treatment journey as evidence of delays or barriers from the manual onboarding process, in line with the impact of complex or inefficient onboarding on adoption of mHealth interventions established in prior work [24]. Despite this hurdle, engagement with the platform was relatively high and stable over time. The presented outcomes do not capture the behavior of individuals who use the platform as a daily alarm without a reporting component, a functionality directly used by other mHealth interventions tested in several settings [2], suggesting that the platform plays a role in promoting adherence for more than half of enrolled patients throughout the duration of treatment. Comparisons with other results are impeded by substantial variation in measures used to gauge “engagement” [25] (eg, qualitative interviews [26], rates of messages received and read [27], and composite engagement indices [28] have previously been used).

Ensuring that users have continued internet access is understood to be important in ensuring widespread adoption of mHealth interventions in similar settings [29]. This study supports this; participants’ access to data influenced their engagement with the platform, as was evident from the increase in the probability of engagement in the period shortly after receiving data top-ups. While the estimated effect was relatively modest in the full sample model, recently receiving data as part of this study is but a noisy signal of users encountering these hard limits to platform usage. As such, any significant effect is a clear indication that mobile data scarcity is a central challenge facing internet-based mHealth interventions in similar settings, which adds to our understanding regarding patient-side costs as an inhibiting factor in mHealth uptake in lower-income settings [30]. Closely related to this are the demonstrated impacts that technical issues and functional limitations can have on engagement. Reminders not being sent or patients not receiving mobile data bundles have been demonstrated to be associated with observable declines in the use of this platform. If usage is associated with improved adherence, as our results suggest that it is, these technical issues may have real consequences for health and well-being. As such, prioritization of system health becomes critical, especially when such interventions are rolled out on a larger scale.

The estimated effect of enrollment on treatment outcomes points to similar mHealth interventions having the potential to significantly impact health and well-being, although the concerns regarding nonrandom selection into the intervention are important caveats. In the context of prior work on the impact of mHealth interventions on tuberculosis treatment, our effects are large. Meta-analysis of 76 studies by Mohamed et al [13] estimated an odds ratio of 1.14 (95% CI 0.99 to 1.30) for the impact of mHealth on treatment success. Estimating our effect as an odds ratio gives 3.08 (95% CI 1.00 to 9.50) for the unadjusted model and 2.40 (95% CI 0.71 to 8.20) for the adjusted model.

### Limitations

It should be acknowledged that human interactions with study participants could not be fully standardized and may have influenced participants’ experiences and responses. Furthermore, due to the iterative design of this study, the experience and engagement of enrolled patients were shaped by the specific features and functionalities of the mHealth platform available at the time of their enrollment.

While results are encouraging, we acknowledge that the comparison of tuberculosis treatment outcomes between enrolled and nonenrolled patients is not causal. Even after adjusting for selected variables to enhance comparability between the groups, there may still be unmeasured differences that these variables do not account for. Similarly, the small sample size limits the statistical power of the analysis, limiting the hypotheses that can be tested using these data. Additionally, although the platform was designed to target specific behavioral barriers, including forgetfulness, present bias, and misperceptions of treatment costs and benefits, the data do not allow us to isolate the contribution of individual mechanisms to the observed outcomes. Our ongoing randomized controlled trial across ten clinics will offer robust and credible impact estimates of this mHealth platform, with an updated study design for further analysis of the mechanisms driving any potential behavioral change. However, the insights gained from the pilot phase remain valuable for both researchers and practitioners, providing important lessons for the design and implementation of future interventions.

### Conclusions

This study contributes to a growing evidence base on the value of mHealth platforms for vulnerable, low-income patient populations. While the benefits of such platforms among higher-income, higher-literacy users are well established, evidence for low-income groups remains limited. Our results add to this discussion by providing early evidence that smartphone-based interventions have a role to play in the fight against disease associated with poverty, even in LMICs. This points to the need for more rigorous identification strategies, including experimental work, in isolating the platform’s independent contribution relative to standard care.

Among our pilot sample, we find encouraging marginal effects of platform enrollment on treatment outcomes, even after adjusting for key observables—results that likely reflect, in part, the iterative, context-sensitive, and theory-driven design of this intervention and the deliberate provision of mobile data to sustain engagement. These design features may prove to be necessary conditions for effectiveness in resource-constrained settings and offer a practical template for similar interventions.

Even when designed to overcome specific barriers, our evidence suggests that the platform is particularly effective among younger patients—a group that is generally underserved by conventional tuberculosis care and faces disproportionately high rates of treatment default. Reaching this population through a low-cost digital intervention is a meaningful contribution in its own right, with the added benefit of potentially freeing clinical resources for patients who require more intensive, in-person support.

## Supplementary material

10.2196/85242 Multimedia Appendix 1 English version of participant consent form administered digitally to all patients during screening.

10.2196/85242 Multimedia Appendix 2 Distribution of patient with tuberculosis’ characteristics by enrollment and smartphone ownership.

## Abbreviations

LMIC: low- and middle-income country
mHealth: mobile health
