# Supplementary figures and images for: Conversational mHealth Platform Designed to Support Tuberculosis Treatment Adherence in Low-Income South African Patients: Pilot Cohort Study
Source: JMIR Form Res. 2026 Jul 8;10:e85242. doi: 10.2196/85242 (PMC13346643; doi:10.2196/85242)

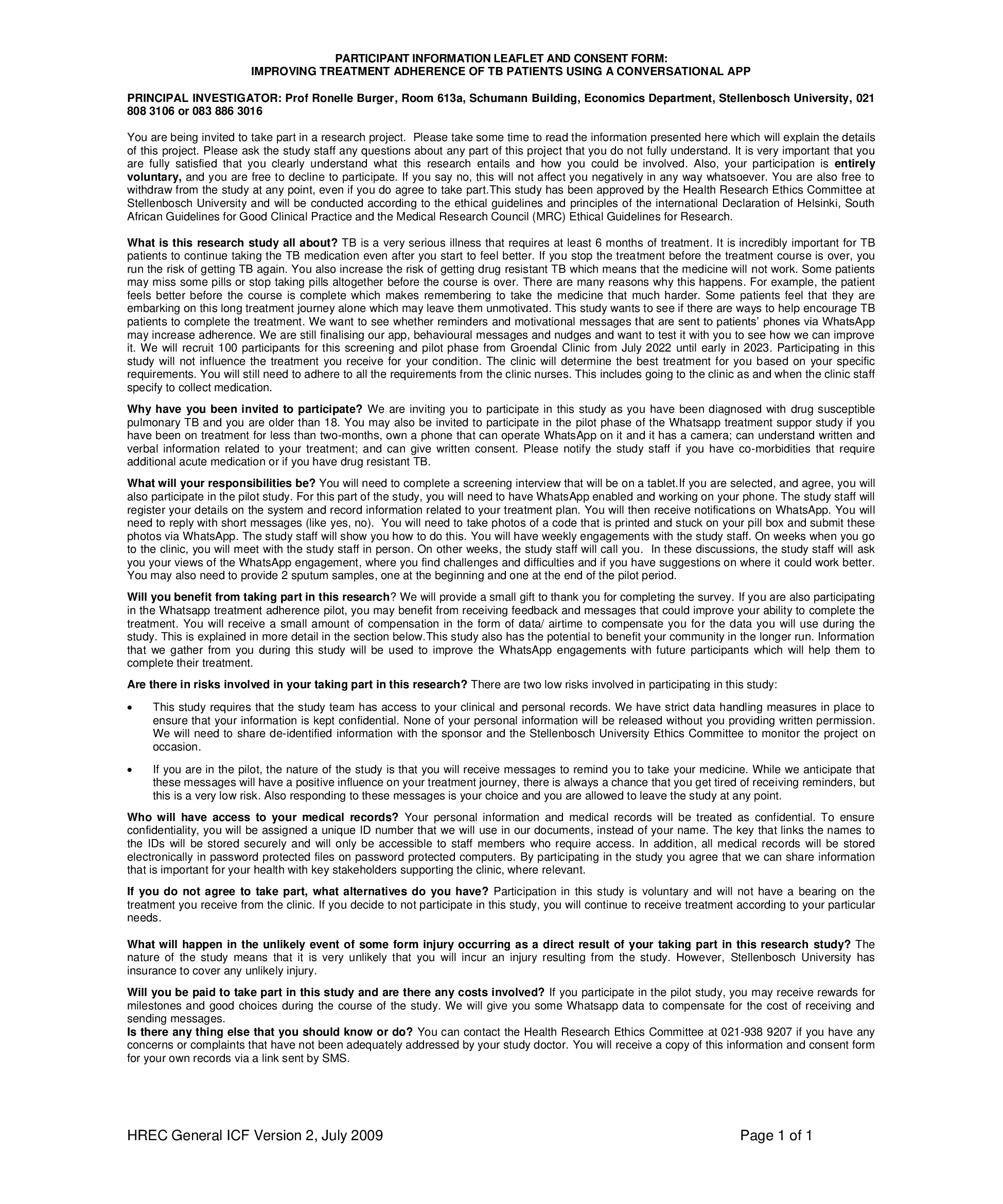

Supplement: Multimedia Appendix 1 — English version of participant consent form administered digitally to all patients during screening. [file formative-v10-e85242-s001.png]
