# Supplementary material for: Conversational mHealth Platform Designed to Support Tuberculosis Treatment Adherence in Low-Income South African Patients: Pilot Cohort Study
Source: JMIR Form Res. 2026 Jul 8;10:e85242. doi: 10.2196/85242 (PMC13346643; doi:10.2196/85242)
Supplement: Multimedia Appendix 2 — Distribution of patient with tuberculosis’ characteristics by enrollment and smartphone ownership. [file formative-v10-e85242-s002.docx]

| **Variable** | **Category** | **Patients not enrolled on platform (n = 102)** | | **Own smartphone & enrolled (n = 42)** |
| --- | --- | --- | --- | --- |
|  |  | **Not own smartphone & not enrolled (n = 95)** | **Own smartphone & not enrolled (n = 7)** |  |
| Gender | Male | 59 | 2 | 22 |
|  | Female | 36 | 5 | 20 |
| Age (years) | 18–29 | 18 | 4 | 11 |
|  | 30–39 | 30 | 1 | 15 |
|  | 40–49 | 20 | 0 | 12 |
|  | 50–59 | 21 | 0 | 4 |
|  | 60+ | 6 | 2 | 0 |
| Education (highest level attained) | Primary or less | 15 | 2 | 4 |
|  | Incomplete secondary | 61 | 3 | 12 |
|  | Completed secondary | 18 | 1 | 24 |
|  | Any tertiary (complete/incomplete) | 1 | 1 | 2 |
| Employment | Employed | 47 | 3 | 27 |
|  | Unemployed, not looking for work | 12 | 2 | 2 |
|  | Unemployed, looking for work | 36 | 2 | 13 |
